# The Semi-Centennial of the Discovery of Anæsthesia

**Published:** 1894-07

**Authors:** 


ARTICLE IV.

### THE SEMI-CENTENNIAL OF THE DISCOVERY OF ANÆSTHESIA.

The year 1844 gave to surgery one of the greatest boons ever conferred upon suffering humanity. The discovery of anæsthesia marked a distinct era, and the credit of it, by universal consent, belongs to dentistry. As in the case of all other great discoveries, a number having claimed to originate it, but the consensus of opinion has awarded to the dentist, Horace Wells, of Hartford, Conn., the honor of first demonstrating it, and his name is enrolled among those highest in the temple of fame as being one of the greatest benefactors of any age. This is the semi-centennial year of its discovery, and dentistry should not miss the opportunity of reminding the world of the debt which it owes to a member of the young profession. Dentists themselves use anæsthetic agents largely in their practice, and they would be recreant to duty and prove themselves but ingrates if they did not in some way mark the occasion by a fitting observance of the anniversary.

The Odontological Society of Pennsylvania took action early in the year, by appointing a committee to take the matter in consideration. The Dental Society of the State of New York, at its annual meeting early in may, appointed a committee to take action upon the part of the dentists of the State. The Connecticut State Dental Society, at its annual meeting, passed resolutions which will be found on another page of this number, and appointed a committee to arrange a semi-centennial celebration, and other societies will doubtless take similar action.

But the subject is too great and too comprehensive in interest to be monopolized by any single body of men. Every dentist throughout the world is concerned in the matter, and should have an opportunity in some way to assist in celebrating an event that must be to him a matter of personal pride. But the time is limited, and whatever is to be done must be done quickly. Half the year has flown without any general organization having been affected, and unless immediate action be taken the most momentous event in our professional history will not receive the attention which its importance demands.

In this emergency we take the liberty to suggest that every dental society in the land, as far as possible, appoint delegates to meet at Old Point Comfort at the time of the annual sessions of the American and the Southern Dental Associations, there to agree upon some concerted plan of action. It is no time for any elements of personality to enter into the consideration of the matter, or for any claims of priority in conception to be urged. Let us as one man unite in honoring ourselves and our profession, by doing honor to the memory of the man who did so much to honor us. We sincerely hope that there will be a unanimity of feeling among us, and that every dental society, especially every State organization, will through its proper officers appoint a committee to meet at Old Point Comfort, Virginia, in August next. We commend the subject to our brother editors of dental journals.—*Dental Practitioner and Advertiser.*

[As chairman of the committee of the New York State Dental Society, Dr. W. C. Barnett will be glad to receive communications from other like committees.]

---

ARTICLE V.

